# Projected Spatial–Temporal Habitat Patterns of the Lady Amherst's Pheasant (*Chrysolophus amherstiae*) Under Climate and Land Use Change

**DOI:** 10.1002/ece3.72457

**Published:** 2025-11-05

**Authors:** Xue Sun, Zexu Long, Jiahao Fang, Sikan Chen, Yue Sun

**Affiliations:** ^1^ School of Biological Science Guizhou Education University Guiyang Guizhou China; ^2^ Guizhou Institute of Forestry Inventory and Planning Guiyang Guizhou China

**Keywords:** biomod, climate change, ensemble modeling, Lady Amherst's pheasant, land use change, multi‐scale

## Abstract

Global climate change and land use change have led to substantial range contractions and shifts. Even more dramatic changes are projected for the future. The Lady Amherst's pheasant is a typical ground‐dwelling bird primarily distributed in China, with very limited flight capabilities, making it more vulnerable to rapid environmental changes. We used a multi‐scale ensemble species distribution modeling approach to model the habitat suitability of the Lady Amherst's pheasant and projected the model to several combinations of SSP‐RCP scenarios and time. We found that the characteristic scale for most variables is relatively large. The ensemble model had an AUC value of 0.98, outperforming individual models. The area of suitable habitat for the Lady Amherst's pheasant is projected to decrease at varying degrees (−2.1% ~ −62.1%) under different scenarios. Dispersal ability only has a little influence on the assessment of suitable habitat loss (−1.5% ~ −60.9%). Loss of suitable habitat mainly occurred in low‐altitude areas, while gain of habitat occurred in medium‐to‐high‐altitude areas. Our results may provide a scientific basis for future conservation strategies of this beautiful pheasant.

## Graphical abstract

A multi‐scale ensemble distribution model was used to evaluate the *Chrysolophus amherstiae* under different climate and land use change scenarios. Habitats of *C. amherstiae* are projected to decrease at varying degrees (−2.1% ~ −62.1%) under different scenarios. Dispersal ability did not significantly affect the habitat change rate evaluation.

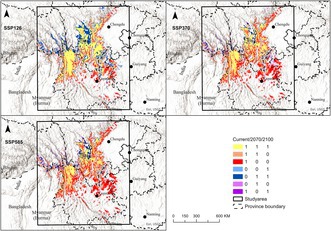

## 1 Introduction

Global climate change, land use, and land cover change are the fundamental drivers of biodiversity loss (Newbold 2018) through suitable habitat range reduction (Powers and Jetz 2019), population decline, and genetic diversity loss (Hu et al. 2021). Human activity is warming the planet at a much faster rate than we have seen in the last 2000 years, with a predicted temperature increase of 1.5°C within the next two decades (Chen et al. 2018). Climate change can increase the frequency and magnitude of extreme weather events, such as prolonged scorching weather, heavy rainfall, and drought. Rapid climate change may aggravate the extinction risk of species that have limited dispersal ability or weak adaptability to a new environment. Land use and human population change are estimated to cause more than 20% species loss across 28% of the terrestrial surface (Newbold et al. 2015). Rising global temperatures will cause areas suitable for growing crops to move to higher latitudes and altitudes, exacerbating the pressure of land use change in these areas, thereby increasing the survival pressure on wildlife (Beniston 2003). Previous studies have mostly focused on the impact of climate change on species' suitable habitats (Li et al. 2010; Chhetri et al. 2021; Wang et al. 2022; Namkhan et al. 2022) and have rarely considered the effect of land use change (Liu et al. 2023).

The Lady Amherst's pheasant ( *Chrysolophus amherstiae* ) is one of the two species in the genus *Chrysolophus* of the family Phasianidae of the order Galliformes; the other one is the golden pheasant ( *Chrysolophus pictus* ). The wild population of *C. amherstiae* is mainly distributed in southwestern China and northeastern Myanmar, and they mainly inhabit mixed coniferous and deciduous broad‐leaved forests (Kang and Zheng 2007). Within the native range area of *C. amherstiae* , high population density, backward social‐economic development, habitat degradation and fragmentation, and poaching have led to a decrease in the population. *C. amherstiae* is listed as Near Threatened in China's Red List of Biodiversity (Zhang and Zheng 2021) and is listed as a class II‐protected species under China's Wild Animal Protection Law. *C. amherstiae* is typically distributed at medium to high altitudes (1500 ~ 4000 m), but it can also be found at low altitudes (800 m; Wu 1984). *C. amherstiae* has limited migration ability; therefore, environmental change can have a great impact on its habitat. Predicting its spatial–temporal habitat pattern under climate and land use change is the foundation for future conservation strategies.

Species distribution models, also called ecological niche models, are a common method used to assess the impact of future environmental changes on species habitats (Elith and Leathwick 2009). Species distribution models use a certain statistical algorithm to establish the functional relationship between species and environmental variables, and then map them to geographic space to obtain the probability of species occurrence or habitat suitability (Guisan and Zimmermann 2000). Many algorithms are available to simulate species‐environment associations, such as generalized linear model, maximum entropy model, random forests model (Elith et al. 2006). When assessing the climate change effects on species distribution using species distribution models, researchers have to make several decisions, such as the selection of variables, statistical methods, and general circulation models, which can lead to large uncertainty in results (Buisson et al. 2010). Among these uncertainty sources, statistical methods contributed the most to the results' uncertainty (Buisson et al. 2010). It is better to integrate several models to get an ensemble model to reduce uncertainty, which may increase practitioners' confidence when making decisions (Araujo and New 2007). Additionally, species respond to different environmental variables at different spatial and temporal scales (Levin 1992). For example, the Mexican spotted owl ( *Strix occidentalis lucida* ) responds strongly to slope at the scale of 200 m, while the optimal scale for the percentage of canopy cover is 1500 m (Timm et al. 2016). Traditionally, the scale of variables is arbitrarily determined by the researcher or by the spatial resolution of variables. Accounting for the scale of variables when modeling habitat can improve model performance and habitat suitability mapping, which is useful for conservation management (Sun et al. 2021). To improve estimation accuracy, we use a multi‐scale ensemble modeling approach to assess the influence of global climate and land‐use change on the habitat of *C. amherstiae* .

## 2 Materials and Methods

### 2.1 Study Area

**FIGURE 1:**
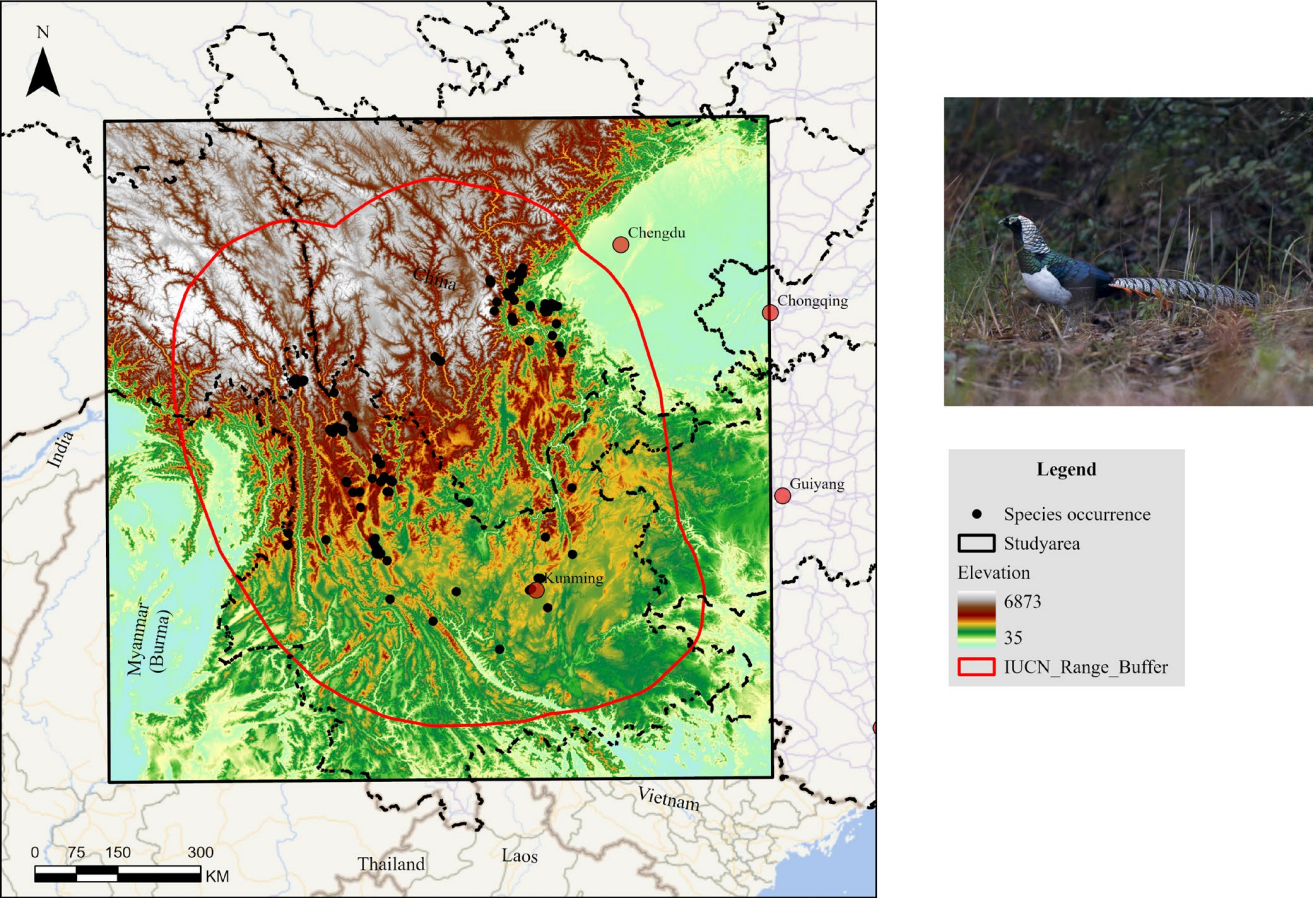
Schematic diagram of the study area. The red polygon indicates the 110 km buffer zone of the IUCN resident range. Photo copyright of *C. amherstiae* Xiaocong Ke.

This study only focused on the native range area of *C. amherstiae* and excluded areas where the species has been introduced (e.g., England). We buffered 200 km of the IUCN range and used the rectangular range as the study area (Figure 1). The geographical range of the study area is 95.594° ~ 106.639° E, 21.836° ~ 32.827° N, the altitude range is 35 ~ 6873 m, and the area of the study area is about 1,270,351 km^2^. The main land cover types in the study area are forest, cultivated land, and grassland. Cultivated land is mainly distributed in low‐altitude areas, grassland is primarily distributed in high‐altitude areas, and forest is between the two.

### 2.2 Species Occurrences

The species occurrences used for modeling come from two sources. (1) We downloaded 688 occurrences from the GBIF (Global Biodiversity Information Facility) between 1979 and 2010; (2) we further collected 15 points by extracting from the literature by searching “Lady Amherst's Pheasant” on CNKI (China National Knowledge Infrastructure) and Google Scholar. To reduce spatial clustering of species points, which could lead to model overfitting and exaggerate model performance indicators (Veloz 2009), we used SDMtoolbbox (Brown and Anderson 2014) to generate a spatial random sample from the occurrence points of *C. amherstiae* . We retained only one occurrence point per 1‐km square grid cell. In addition, we removed some potentially erroneous occurrence points if they met any of the following conditions: (1) Points outside the species' IUCN range polygon; (2) Points where the elevation was not within the IUCN Red List designated elevation range (after adding a 100 m buffer zone); (3) Points where the land cover type did not correspond to the habitat type (forest and shrubland) specified by the IUCN Red List. After these treatments, 138 occurrence points remained for modeling.

### 2.3 Environmental Predictors

We used climate factors, topographic factors, vegetation factors, and anthropogenic disturbance factors to characterize the habitat requirements of *C. amherstiae* (Li et al. 2010; Wang et al. 2022), as detailed in Table 1. The climate factors include annual average temperature, standard deviation of monthly average temperature, average temperature of the hottest month, average temperature of the coldest month, total annual precipitation, precipitation in the wettest month, precipitation in the driest month, and seasonal variation in precipitation. The climate factors were sourced from the CHELSA V2.1 Climatologies at High Resolution for the Earth Land Surface Areas (CHELSA V2.1, https://chelsa‐climate.org/downloads/). We used the mean values of the 1981–2010 climate variables to represent the current conditions.

**TABLE 1:** Variables abbreviation, description, sources, their scales of effect, and *t*‐ and *p*‐values from the univariate scaling process.

Variable	Description	Source	Scale (km)	*p*
Elevation	Mean elevation	EarthEnv global 1‐km topography, https://www.earthenv.org/topography	8	2.42 × 10^−3^
Slope	Slope		7	5.35 × 10^−16^
tri	Terrain ruggedness index		8	8.39 × 10^−13^
Northness	Northness = sin (slope) * cos (aspect)		10	2.70 × 10^−10^
annualtem	Mean annual air temperature	CHELSA (Climatologies at high resolution for the earth's land surface areas) v2.0.1, 1 km. https://chelsa‐climate.org/	7	5.47 × 10^−7^
temvar	Temperature seasonality		—	—
tem_warmmon	Mean daily maximum air temperature of the warmest month		6	2.33 × 10^−13^
tem_coldmon	Mean daily maximum air temperature of the coldest month		8	1.49 × 10^−3^
annualprec	Annual precipitation amount		—	—
prec_wetmon	Precipitation amount of the wettest month		—	—
prec_drymon	Precipitation amount of the driest month		—	—
prec_var	Precipitation seasonality		1	1.04 × 10^−3^
Forest	Percentage of forest area within a moving window with a certain radius	Future global land datasets with a 1‐km resolution based on the SSP‐RCP scenarios, https://zenodo.org/records/4584775	7	2.55 × 10^−42^
Cropland	Percentage of cropland area within a moving window with a certain radius		10	1.90 × 10^−22^
Footprint	Human footprint index	Last of the Wild Project, Version 3 (LWP‐3): 2009 Human Footprint, https://www.earthdata.nasa.gov/data/catalog/sedac‐ciesin‐sedac‐lwp3‐hf‐2009‐2018.00	1	4.15 × 10^−2^

Four topographic factors, elevation, slope, northness, and terrain ruggedness index, were used. The topographic data were sourced from the EarthEnv topographic variables (Amatulli et al. 2018), with a spatial resolution of 1 km. The vegetation factors include the proportion of forest area and the proportion of cultivated land within a specific range. Land use and cover data were sourced from a global land projection at a 1‐km resolution (Chen et al. 2022). We chose this dataset because it includes predictions of land use and cover under current and future Shared Socioeconomic Pathways (SSPs), and the scenarios align with the future climate variables. We used the human footprint index to represent anthropogenic disturbance. This index is a composite measure of human activity impacts on the environment, integrating eight variables such as built‐up environments, population density, electric power infrastructure, crop lands, roads, and railways (Venter et al. 2016). The human footprint index has a spatial resolution of 1 km, and we used the data from 2009.

### 2.4 Variables in Future Scenarios

This study investigates the habitat conditions of *C. amherstiae* under different combinations of climate change and land use change scenarios in the future. We considered two time periods (2041–2070, 2071–2100) and three Shared Socioeconomic Pathways with Representative Concentration Pathways (SSP‐RCPs). The three SSP‐RCPs are SSP126, SSP370, and SSP585. SSP126 represents the combination of SSP1 and RCP2.6, indicating a low level of greenhouse gas emissions and a sustainable development path. SSP126 was the combination of SSP3 and RCP 7.0, representing a medium level of greenhouse gas emissions and a regional rivalry development scenario. SSP585 is the combination of SSP5 and RCP8.5, representing continuously high‐rising carbon emissions. SSP126 represents the most optimistic estimate for future development, SSP585 represents the upper limit of the scenario range described in the literature, and SSP370 falls in the upper‐middle range of the two scenarios. The climate variable projections are also sourced from CHELSA. For each future scenario, we used the projected values from three global climate models (GCMs): GFDL‐ESM4, UKESM1‐0‐LL, and MPI‐ESM1‐2‐HR. These three GCMs are the top three models recommended by the ISIMIP3b protocol. Future land use projection was from a global land projection with a 1‐km resolution under socio‐climatic scenarios (Chen et al. 2022). The future land use scenarios are the same as the climate change scenarios.

In ArcGIS Pro 3.2 (ESRI 2019), all variables were clipped and projected to the study area boundary, and then uniformly resampled to a 1 km grid size. To identify the effect scale of each variable, we created multi‐scale variables. Using the *focal* function in the R package “terra” (Hijmans et al. 2025), we calculated the mean value of continuous variables and the percentage of categorical variables within moving windows of different radii, and assigned them to the central grid cell. The window radii tested included 1, 2, 3, 4, 5, 6, 7, 8, 9, and 10 km.

### 2.5 Generating Multi‐Scale Species Distribution Models

First of all, we identify the characteristic scale for each variable by performing *t‐tests* between values at the presence and pseudo‐absence points for each variable at each scale (Shirk et al. 2018). We randomly selected 20,000 pseudo‐absence points from the entire study area. For each variable, we only retained scales with *p*‐values less than 0.05 and identified the scale of effect with the lowest *p*‐value (Table 1). To alleviate the issue of multicollinearity among environmental variables, we ran pairwise Pearson's correlations between all remaining variables, and we retained the variable with the lower *p*‐value if correlations greater than |0.7|are present (Figure S1). With this final multi‐scale variable set, we used an ensemble modeling approach (Araujo and New 2007) to model the distribution of *C. amherstiae* . We implemented ensemble modeling using the “biomod2” package (Thuiller et al. 2025) in R software v.4.5.1 (R Core Team 2025). We randomly selected 20,000 pseudo‐absence points (excluding presence points) to represent the available environment in the study area. We selected three pseudo‐absence sets, each with 20,000 points, to mitigate the uncertainty that one pseudo‐absence set may cause. In this study, we selected five commonly used algorithms, which are usually demonstrated to have good model performance (Valavi et al. 2021). These five algorithms are generalized linear models (GLM), generalized additive models (GAM), maximum entropy models (MaxEnt), random forest models (RF), and extreme gradient boosting training models (XGBOOST). The model option for each algorithm was set as “bigboss”, whose default parameter values are predefined by the “biomod2” team. We used an AUC‐weighted mean method to ensemble all models that have an AUC value higher than 0.7 (Marmion et al. 2009). We also created binary maps from the ensemble model based on the threshold that maximizes the sum of model sensitivity and specificity (Liu et al. 2013).

We assessed the predictive performance of models with a random 5‐fold cross‐validation procedure and repeated this procedure 3 times. We used three indices, the Area Under Receiver Operating Characteristic Curve (AUC, Hanley and McNeil 1982), the True Skill Statistic (TSS, Allouche et al. 2006), and the Boyce index (Boyce et al. 2002), to reflect model performance. AUC is a threshold‐independent index and ranges from 0 to 1, with higher values indicating a stronger ability of the model to discriminate presence from background points. TSS is a threshold‐dependent index and is not influenced by species prevalence. TSS ranges from −1 to 1, with a value higher than 0.4 indicating good model performance. The Boyce index is suitable for evaluating presence‐only models and ranges from 0 to 1.

### 2.6 Projecting Future Habitat

We projected the final ensemble model for each future time step, 2070 and 2100, for each SSP‐RCP and for each of the three GCMs (Chen et al. 2022). For each SSP‐RCP scenario and time step, we averaged the habitat suitability projections across the three GCMs. The current and future ensemble projections were not rescaled and divided into five bins for comparison. We also divided the continuous ensemble suitability maps into binary maps (suitable habitats vs. non‐habitats) using the threshold that maximizes the sum of test sensitivity and specificity. In creating the binary maps from the future suitability maps, we used the same threshold value as for the current time.

Dispersal ability is a key factor affecting the ability of species to respond to environmental change (Gouveia et al. 2016). There is little research focusing on *C. amherstiae* dispersal patterns, while it is considered to have very limited dispersal ability (Li et al. 2010). We considered two dispersal scenarios under which to identify the species' suitable habitats. Firstly, we assumed *C. amherstiae* can disperse to all potential habitats as a “perfect” dispersal scenario. Secondly, we assumed *C. amherstiae* has limited dispersal abilities and created a ~110 km buffer as “limited dispersal scenarios” (Namkhan et al. 2022). We also created time‐step maps describing species range shifts across multiple time periods. Time‐step maps detail the step‐wise expansions and contractions of *C. amherstiae* distribution through time‐steps. We also calculated the loss, gain, net change, and turnover of suitable habitats for each scenario and time period. Suitable habitat turnover is calculated as (habitat gain + habitat loss)/(current habitat + habitat gain) (Luo et al. 2015).

## 3 Results

**FIGURE 2:**
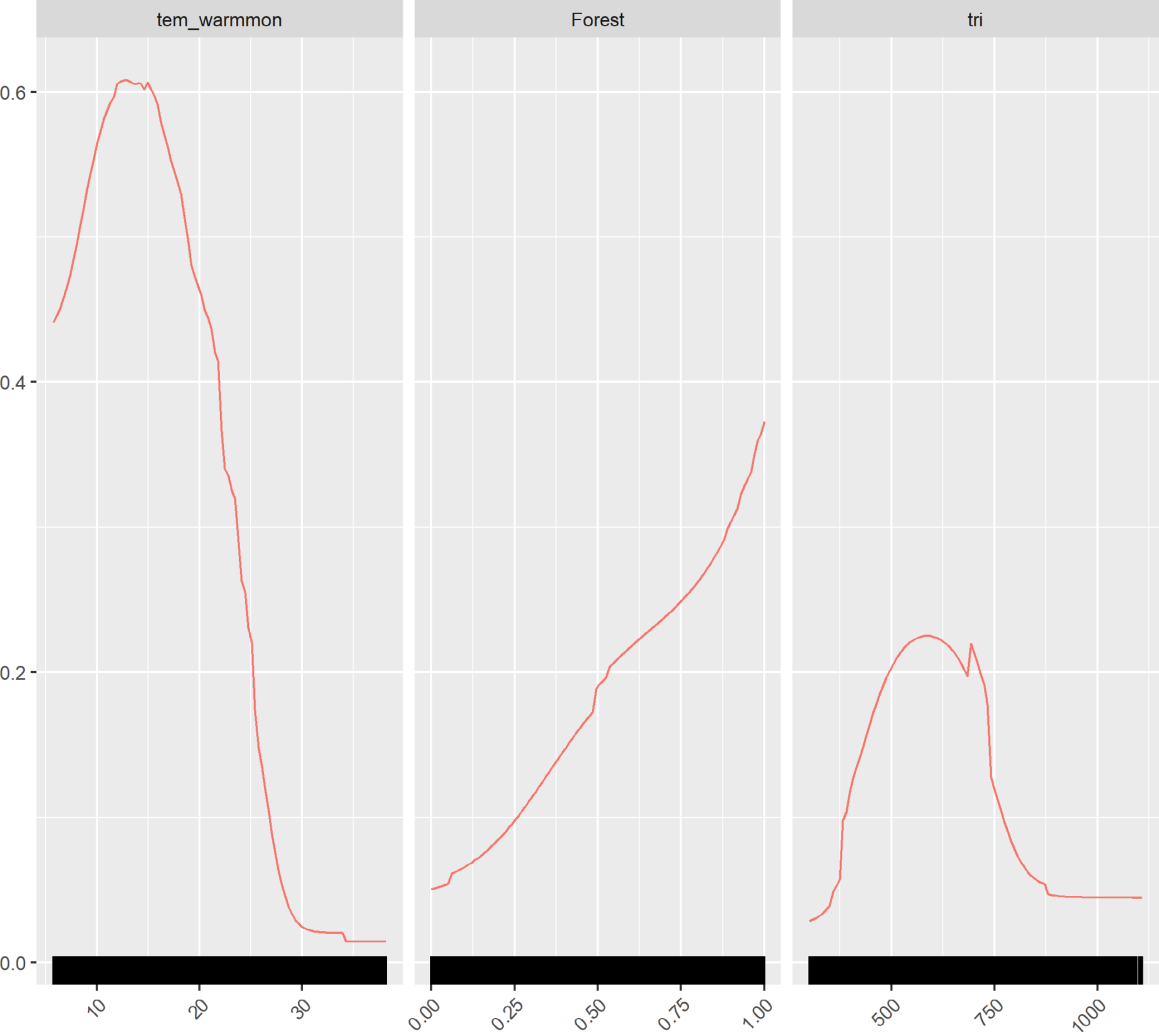
Response curve of the three most important variables of the ensemble model for *C. amherstiae*: tem_warmmon, mean daily maximum air temperature of the warmest month; Forest, percentage of forest area within a 7 km radius window; tri, terrain ruggedness index.

**FIGURE 3:**
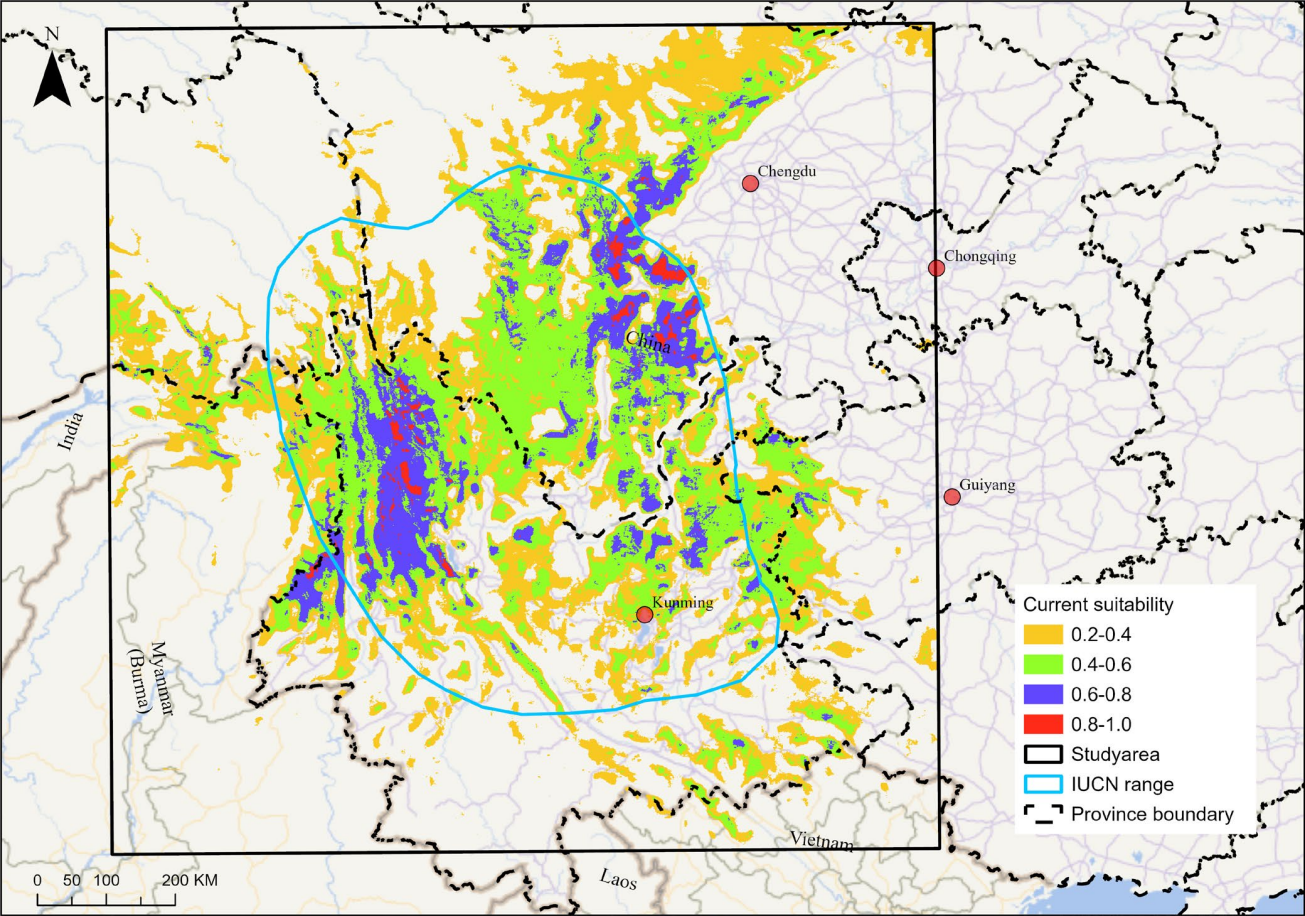
Current habitat suitability map of *C. amherstiae* predicted by the ensemble model. The light blue polygon represents the IUCN resident range area of *C. amherstiae* . The original continuous habitat suitability value was divided into 5 bins using the equal interval method.

*t*‐tests indicate that four variable values do not significantly differ between species occurrences and backgrounds (Table 1). Paired correlation plots showed that four variables have high correlation values with other variables and were screened (Figure S1). Eight variables, percentage of cropland (Cropland), human footprint index (footprint), percentage of forest (Forest), northness (northness), precipitation seasonality (perc_var), slope (slope), temperature of the warmest month (tem_warmmon), terrain ruggedness index (tri), were finally used in the ensemble model. The characteristic scale for the human footprint index and precipitation seasonality is at 1 km, while other variables' optimal scales are relatively large (≥ 6 km). The individual SDMs had average AUC values of 0.82, 0.87, 0.88, 0.92, and 0.86 for the GAM, GLM, MaxEnt, RF, and XGBOOST, respectively (Figure S2). All 150 models have AUC values higher than 0.7. The AUC‐based weighted mean ensemble model has an AUC value of 0.98, indicating excellent performance of the model in discriminating occurrence from background points. The TSS and Boyce indices also indicated excellent model performance of the ensemble model with values of 0.87 and 0.99, respectively. Habitat suitability for *C. amherstiae* was mainly determined by the temperature of the warmest month (tem_warmmon), percentage of forest (Forest), and terrain ruggedness index (tri), while other variables had weaker relationships (Table S1). Habitat suitability had unimodal relationships with variable tem_warmmon and tri, while it had a positive relationship with Forest (Figure 2). The habitat suitability map showed areas of high habitat suitability in western Yunnan, northeastern Yunnan, and central Sichuan (Figure 3), and there is only a small proportion of low‐to‐medium suitability (0.2–0.6) areas in Myanmar (Figure 3). See Figure S3 for future habitat suitability maps under different SSP‐RCP scenarios (SSP126, SSP370, and SSP585) and time periods (2041–2070, 2071–2100). By comparing the habitat suitability bins between current and future scenarios, we found that the percentage of medium‐to‐high suitable habitats (habitat suitability > 0.4) generally declined (except for SSP126), while the percentage of lower suitability (habitat suitability < 0.4) habitats increased (Table 2). It showed a similar trend when constrained comparison in the 110 km‐buffer area of the IUCN range (Table S3). The threshold that maximized the sum of the ensemble model's test sensitivity and specificity is 0.427, which is used as a cutoff to binarize the habitat suitability map into suitable habitat and non‐habitat. There is an estimated 248,161 km^2^ of suitable habitat in the study area, of which 189,492 km^2^ falls within the IUCN range.

**TABLE 2:** Percent of the study area in each *C. amherstiae* habitat suitability bin for the current and future time periods (2070 and 2100) under three SSP‐RCP scenarios (SSP126, SSP370, and SSP585).

Suitability class	Current	2070			2100		
		SSP126	SSP370	SSP585	SSP126	SSP370	SSP585
0.0–0.2	67.55%	63.68%	70.98%	72.25%	63.11%	78.31%	80.28%
0.2–0.4	15.02%	18.99%	15.83%	15.21%	19.60%	13.36%	12.79%
0.4–0.6	12.28%	11.03%	8.73%	8.39%	10.94%	5.96%	5.08%
0.6–0.8	4.74%	5.51%	4.06%	3.80%	5.58%	2.24%	1.76%
0.8–1.0	0.41%	0.80%	0.40%	0.35%	0.77%	0.14%	0.09%

**FIGURE 4:**
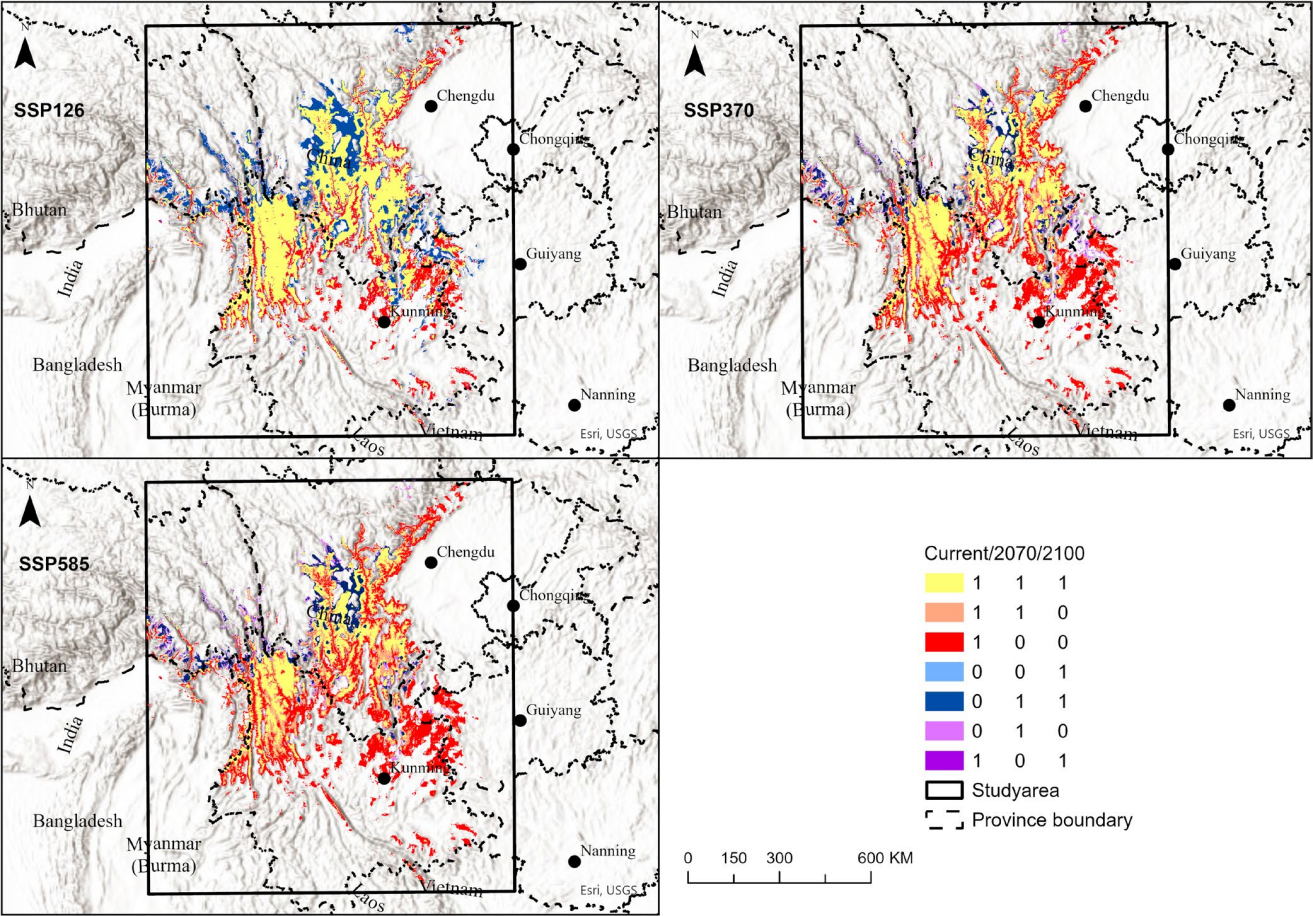
Time step maps of suitable habitat from the current time to 2070 and 2100 under the SSP126, SSP370, and SSP585 scenarios. Bright yellow regions indicate suitable habitat throughout the entire time periods. Blue regions indicate areas of expansion, red regions indicate areas of contraction, and purple regions indicate areas of momentary fluctuations among the three times.

From the current to 2070, 2.1%, 25.7%, and 29.7% of suitable habitat will be lost for the SSP126, SSP370, and SSP585 scenarios, respectively. From the current to 2100, 2.1%, 53.8%, and 62.1% of suitable habitats will be lost for the SSP126, SSP370, and SSP585 scenarios, respectively (Table 3). If we constrain future habitat within the 110 km buffer of the IUCN range, the trend of suitable habitat change is similar to the perfect dispersal scenario, with a slightly smaller percentage of loss (Table S3). The area of suitable habitat that was stable through time varied across future SSP‐RCP scenarios; higher levels of greenhouse gas emissions cause a smaller area of stable habitat (Table 3). The time‐step maps of suitable habitats showed that loss of suitable habitat occurred mainly in western Guizhou, eastern and southeastern Yunnan, and northern Sichuan (orange and red colors in Figure 4). Gain of habitat occurs in the northwest of the study area (dark blue area in Figure 4), where high elevation occurs. We further calculated the resilient areas to climate and land cover change (bright yellow areas in Figure 4), which are suitable habitats from the current to 2100. There are 162,687 km^2^, 90,874 km^2^, and 69,798 km^2^ resilient areas for SSP126, SSP370, and SSP585 scenarios, respectively.

**TABLE 3:** Projection of total area, net change, area gain, area loss, and area turnover of suitable habitat for *C. amherstiae* under the perfect dispersal scenario and current and future climate and land use change scenarios.

	Current	2070			2100		
		SSP126	SSP370	SSP585	SSP126	SSP370	SSP585
Total area (km^2^)	248,161	243030	184,298	174,505	243,038	114,597	94,105
Net percent change (%)		−2.1	−25.7	−29.7	−2.1	−53.8	−62.1
Area Stable (km^2^)		174,565	143,715	132,471	174,138	91,447	69,964
Area gain (km^2^)		68,465	40,583	42,034	68,900	23,150	24,141
Area loss (km^2^)		73,596	104,446	115,690	74,023	156,714	178,197
Area turnover (km^2^)		44.9%	50.2%	54.4%	45.1%	66.3%	74.3%

## 4 Discussion

This study used a multi‐scale ensemble species distribution modeling approach to establish the relationship between *C. amherstiae* and the environment, predicting the current habitat suitability, and forecasting habitat distribution under different future time periods, climate change, and land use change scenarios. We improved species distribution modeling strategies, enhancing the model's performance and providing a scientific basis for formulating conservation strategies for *C. amherstiae* .

We found that the optimal scale for most variables included in the model is between 6 and 7 km, indicating that *C. amherstiae* responds to environmental variables over a more extensive spatial range. In contrast, previous studies on pheasant distribution modeling typically adopted a spatial scale of 1 km for all variables (Li et al. 2010; Wang et al. 2022). They selected such a scale mainly because the resolution of the environmental covariate is 1 km (e.g., WorldClim bioclimate data). The predictive capability of the ensemble model showed a significant improvement over single‐algorithm models, and all three evaluation metrics indicated an excellent model performance. Wang et al. (2022) used the MaxEnt model, with an AUC value of 0.94 and a TSS value of 0.28, to predict the current suitable habitat distribution of *C. amherstiae* . Due to its ease of use and rich output, the MaxEnt model is one of the most commonly used algorithms in species distribution modeling (Elith et al. 2011). MaxEnt model settings may have a large influence on predictions; the default setting is usually not the best for all species. Using the MaxEnt model based on the default setting may lead to less rigorous results, impairing decision‐making (Anderson and Gonzalez Jr. 2011; Muscarella et al. 2014).

The variable importance showed that the mean temperature of the warmest month (tem_warmmon) contributes the most to the habitat suitability of *C. amherstiae* . However, the study by Wang et al. (2022) suggests that seasonal temperature variability is the most significant contributing factor. In our research, seasonal temperature variability did not enter the model because its mean value showed no significant difference between presence and background points. This discrepancy may be due to differences in variable selection methods. Wang et al. (2022) determined which variables were to be retained based on their contribution levels in a univariate MaxEnt model. When the average temperature of the hottest month exceeds 15°C, habitat suitability decreases as temperature rises. *C. amherstiae* is accustomed to living in mid‐to‐high altitude areas, and its physiological structure has adapted to low‐temperature environments. Rising summer temperatures may lead to heat stress. If low habitat connectivity makes it difficult for the species to migrate to higher altitude areas, it could result in their disappearance from lower altitude regions.

Our study, based on an ensemble model, estimated that under current environmental conditions, the area of suitable habitat for *C. amherstiae* is 248,161 km^2^, which is higher than the 215,569 km^2^ estimated by Wang et al. (2022). A comparison of the prediction maps between the two studies reveals that the prediction map of Wang et al. (2022) contains more detail, such as relatively scattered suitable habitat pixels in peripheral edge areas. This is because the characteristic scale of variables used in our study is relatively large, resulting in a coarser spatial pattern of the variables. We believe that smoother maps may be closer to the actual condition. The prediction map based on multi‐scale variable models is smoother, and the results are more conducive to formulating conservation actions (Sun et al. 2021). If landscape indices are calculated based on predictions with excessive detail, it could exaggerate the degree of habitat fragmentation assessment. For more reliable fragmentation estimation, it is recommended to consider habitat connectivity within the area that species can disperse (Zeller et al. 2021).

Understanding *C. amherstiae's* range dynamics during the last several decades is crucial for evaluating the model's relevance and for interpreting future risk accurately. Little information is available to make such an analysis across the whole range, even if it is believed that *C. amherstiae's* range has contracted due to poaching, habitat loss, and fragmentation (Kang and Zheng 2007). We downloaded *C. amherstiae's* occurrence data between 1971 and 2025 from the GBIF and divided it into two periods (1971–2010 vs. 2011–2025). Elevation at occurrence locations was extracted and used to draw a density plot (Figure S4). The mean elevation for the period 1971–2010 and 2011–2025 is 2065 and 2275 m, respectively, which may indicate that *C. amherstiae's* range has shifted. We also created a 95% minimum convex polygon (MCP) for the two time‐period occurrence points and calculated their overlap rate. 90% of the 2011–2025 MCP and 86% of the 1971–2010 MCP overlap, which means the distribution range of *C. amherstiae* has contracted. There are few studies on the effect of climate or land use change on *C. amherstiae* habitat. (Wang et al. 2023) evaluated the climate change effect on 47 species of Galliformes; in their evaluation, 36.2% net habitat loss of *C. amherstiae* is projected under the SSP585 scenario, which is much less than our evaluation (i.e., 62.1%). They only considered the climate change effect and ignored the effect of land use change. Furthermore, two studies, Wang et al. (2023) and our study, used different thresholds to binarize model predictions (10‐percentile threshold vs. TSS maximization threshold). We found that under any scenario, the total area of suitable habitat for *C. amherstiae* will decrease to varying degrees in the future, with greater declines corresponding to higher emission levels. In the SSP126 scenario, the total suitable habitat area changes by −2.1%, which is relatively low, whereas the proportion of suitable habitats turnover is relatively high (44.9%), as the area gain and area loss are high. The area of newly gained suitable habitats (i.e., 68,465 km^2^) is estimated solely based on environmental suitability; whether *C. amherstiae* can disperse to these environmentally suitable areas requires further consideration of habitat connectivity and the species' dispersal ability. Due to the lack of research on dispersal ability, it is difficult to make a robust estimation. If landscape connectivity is low, the area of suitable habitat in future scenarios is likely to be less than the estimates in this study. The loss of suitable habitat mainly occurred in the southwestern and northeastern parts of the study area, which are at relatively lower altitudes (Figure 4). In contrast, the newly gained habitats are primarily located in the northwestern part of the study area.

The assessment of our study is based on the assumption that the association between species and the environment does not change over time, without considering the adaptation of *C. amherstiae* to the environment. Future studies can achieve more accurate assessments by integrating mechanistic and correlative species distribution models (Kearney et al. 2010). We excluded occurrence points in habitats not classified as forest or shrubland per IUCN Red List criteria, which is a restrictive criterion. Our model may underestimate future habitat range, as *C. amherstiae* may show flexibility in the exploitation of resources and use other habitat types (e.g., van Toor et al. 2017).

## 5 Conclusions and Management Implications

Our results reveal the negative effects of global climate change and land use change on *C. amherstiae* across China and Myanmar. The habitat range will decrease at varying degrees (−2.1% ~ −62.1%) depending on future RCP‐SSP scenarios. The predicted habitat loss mainly occurred in Southeast Yunnan, East Yunnan, and West Guizhou. We advocate extending the current protected area network to cover more suitable habitats and maintain/restore habitat connectivity between areas predicted to be lost in the future. It is also significant to strengthen law enforcement and public education to reduce the poaching of *C. amherstiae* . In addition, we also encourage the public to report *C. amherstiae* sightings through platforms like eBird or the Bird Report Center of China, which is helpful for monitoring and assessing habitat range dynamics.

## Author Contributions

**Xue Sun:** conceptualization (equal), software (lead), writing – original draft (lead). **Zexu Long:** conceptualization (equal), formal analysis (supporting), methodology (supporting), project administration (lead), validation (equal), writing – review and editing (equal). **Sikan Chen:** data curation (equal), writing – review and editing (equal). **Jiahao Fang:** validation (equal), visualization (lead). **Yue Sun:** methodology (supporting), writing – review and editing (equal).

## Conflicts of Interest

The authors declare no conflicts of interest.

## Supporting information


**Appendix S1:** ece372457‐sup‐0001‐Appendix.docx.

## Data Availability

All code and data are publicly available at figshare. Doi: 10.6084/m9.figshare.29093711.
