# Supplementary material for: Projected Spatial–Temporal Habitat Patterns of the Lady Amherst's Pheasant (Chrysolophus amherstiae) Under Climate and Land Use Change
Source: Ecol Evol. 2025 Nov 5;15(11):e72457. doi: 10.1002/ece3.72457 (PMC12588769; doi:10.1002/ece3.72457)
Supplement: Supplementary file 1 — Appendix S1: ece372457‐sup‐0001‐Appendix.docx. [file ECE3-15-e72457-s001.docx]

Appendix for

Projected spatial-temporal habitat patterns of the Lady Amherst’s pheasant (*Chrysolophus amherstiae)* under climate and land use change

Xue Sun^1^, Zexu Long^1*#^, Jiahao Fang^1^, Sikan Chen^2^, Yue Sun^1^

^1^ School of Biological Science, Guizhou Educational University, 115 Gaoxin Road, Wudang District, Guiyang, Guizhou, 550018

^2^ Guizhou Institute of Forestry Inventory and Planning

, Guiyang, Guizhou, 550003

* Authors contribute equally

# Author to whom correspondence should be addressed

Xue Sun, sunxue0320@gznc.edu.cn

Jiahao Fang, [fangjh6643@gmail.com](mailto:fangjh6643@gmail.com)

Sikan Chen, sikanchen@foxmail.com

Zexu Long, longzexu1990@gmail.com

Yue Sun, sy1028sy@163.com

Contents of this file:

Table S1. Variable importance of the ensemble model.

| Variable | Importance value |
| --- | --- |
| tem_warmmon | 0.632 |
| Forest | 0.442 |
| tri | 0.201 |
| footprint | 0.062 |
| Corpland | 0.056 |
| prec_var | 0.023 |
| northness | 0.022 |
| slope | 0.015 |

Note: tem_warmmon, mean daily maximum air temperature of the warmest month; Forest, percentage of forest area within 7km-radius window; tri, terrain ruggedness index; footprint, human footprint index; Corpland, percentage of cropland area within 10km-radius window; prec_var, precipitation seasonality; northness, northness; slope, terrain slope.

Table S2. Percent of the 110-km IUCN range buffer area in each C. amherstiae habitat suitability bin for the current and future time steps (2070 and 2100) under three SSP-RCP scenarios (SSP126, SSP370, and SSP585).

| Suitability bin | Current | 2070 | | | 2100 | | |
| --- | --- | --- | --- | --- | --- | --- | --- |
|  |  | SSP126 | SSP370 | SSP585 | SSP126 | SSP370 | SSP585 |
| 0.0-0.2 | 47.32% | 43.67% | 52.71% | 55.22% | 42.81% | 63.79% | 67.43% |
| 0.2-0.4 | 21.08% | 24.75% | 23.11% | 21.72% | 25.71% | 20.66% | 19.58% |
| 0.4-0.6 | 21.65% | 19.33% | 15.48% | 14.95% | 19.17% | 10.90% | 9.36% |
| 0.6-0.8 | 9.14% | 10.67% | 7.91% | 7.41% | 10.79% | 4.38% | 3.47% |
| 0.8-1.0 | 0.81% | 1.58% | 0.80% | 0.70% | 1.52% | 0.27% | 0.17% |

Table S3. Projection of total area, net change, area gain, area loss, and area turnover of highly suitable *C. amherstiae* habitats under the limited dispersal (within 110-km IUCN range buffer) scenario and current and future climate and land use change scenarios

|  | Current | 2070 | | | 2100 | | |
| --- | --- | --- | --- | --- | --- | --- | --- |
|  |  | SSP126 | SSP370 | SSP585 | SSP126 | SSP370 | SSP585 |
| Total area(km^2^) | 230377 | 226935 | 172858 | 164255 | 226758 | 109001 | 68247 |
| Net percent change(%) |  | -1.5 | -25.0 | -28.7 | -1.6 | -52.7 | -60.9 |
| Area Stable(km^2^) |  | 166183 | 137729 | 127260 | 165424 | 88680 | 68247 |
| Area gain(km^2^) |  | 60752 | 35129 | 36995 | 61334 | 20321 | 21892 |
| Area loss(km^2^) |  | 64194 | 92648 | 103117 | 64953 | 141697 | 162130 |
| Area turnover(km^2^) |  | 42.9% | 48.1% | 52.4% | 43.3% | 64.6% | 72.9% |


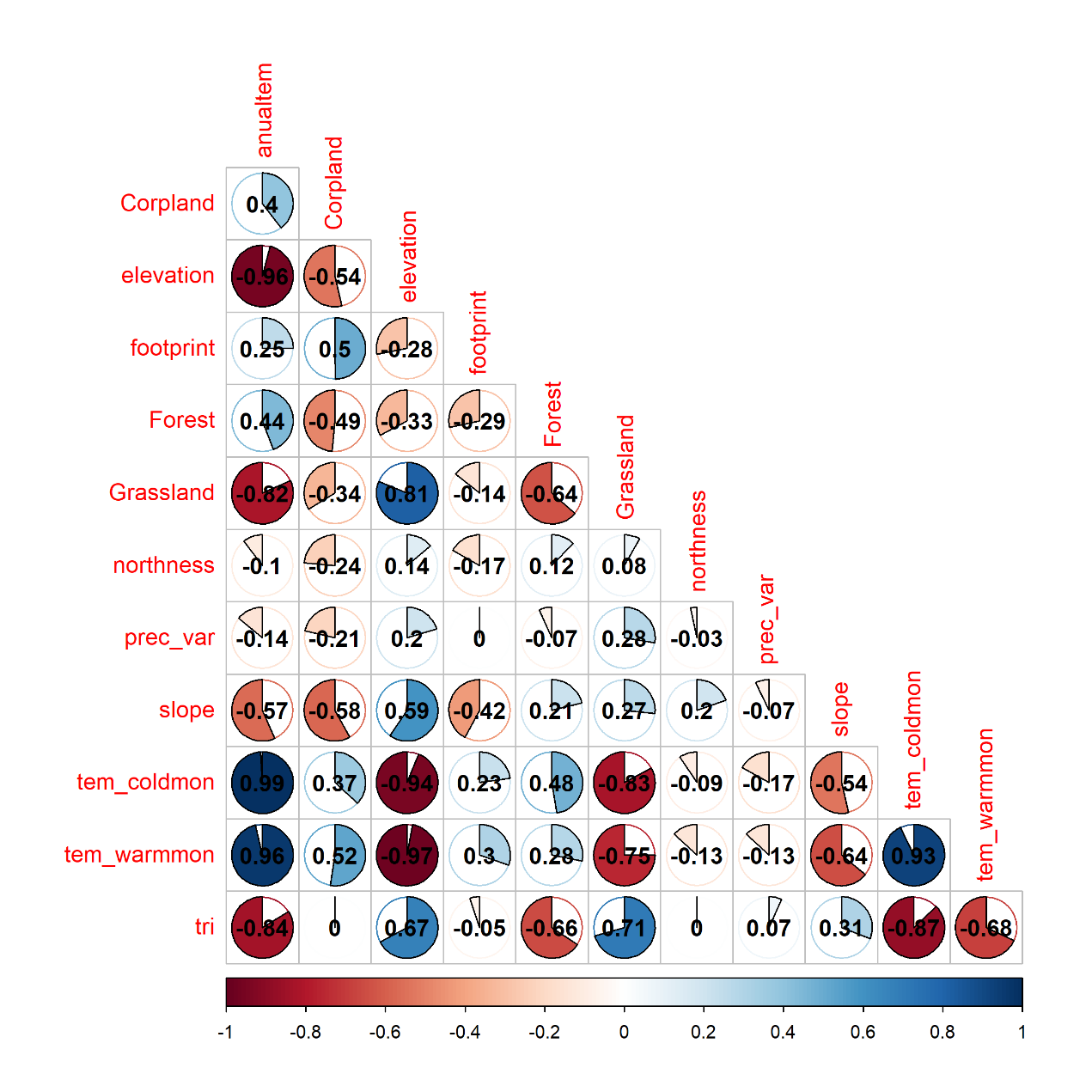


Figure S1. Pearson correlation matrix plot between environmental variables.


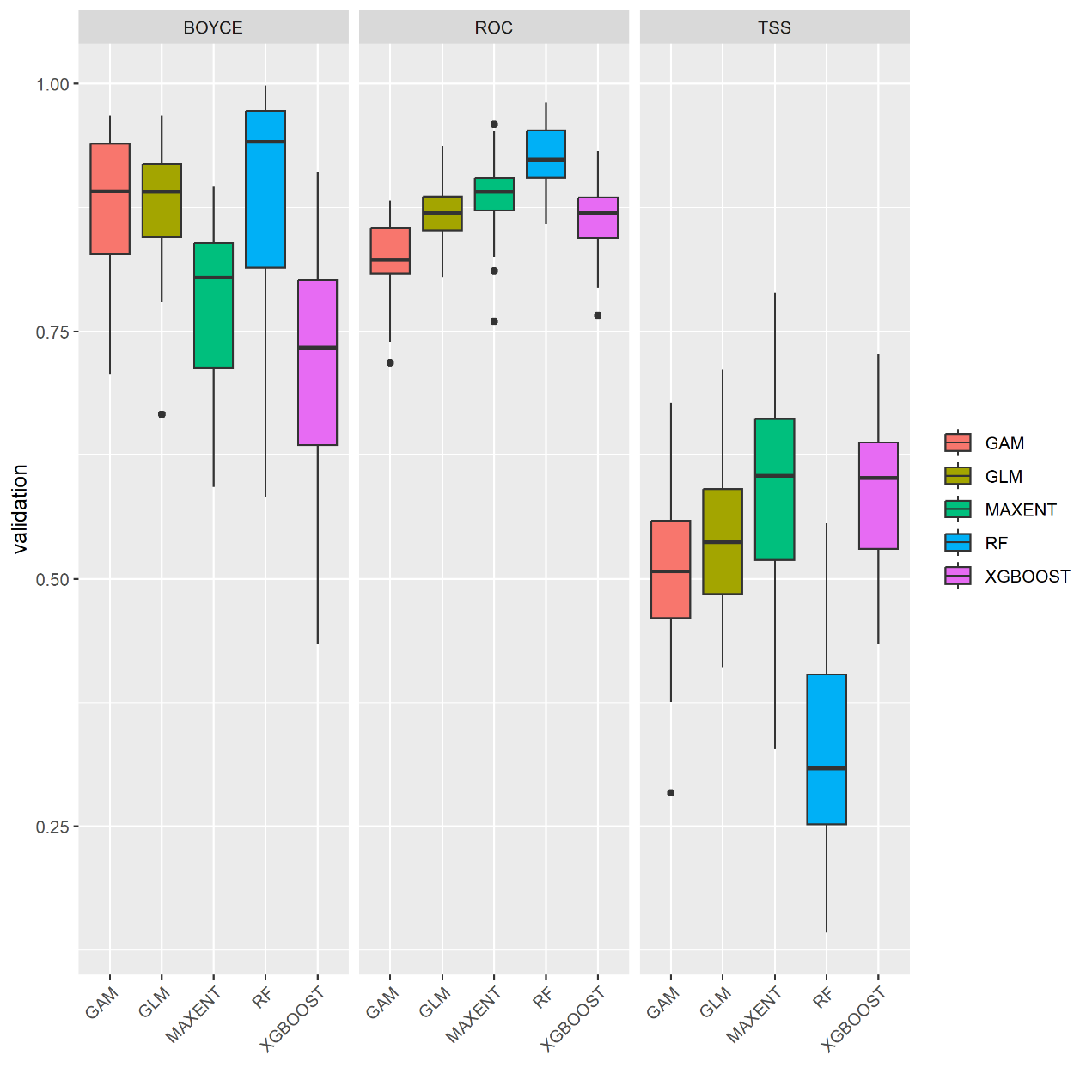


Figure S2. Boxplot of five single-algorithm models under three model metrics. GAM, generalized additive model; GLM, generalized linear model; MAXENT, maximum entropy model; RF, random forests model; XGBOOST, extreme gradient boosting training model. BOYCE, the boyce index; ROC, the Area Under Receiver operating characteristic curve; TSS, the true skill statistic.


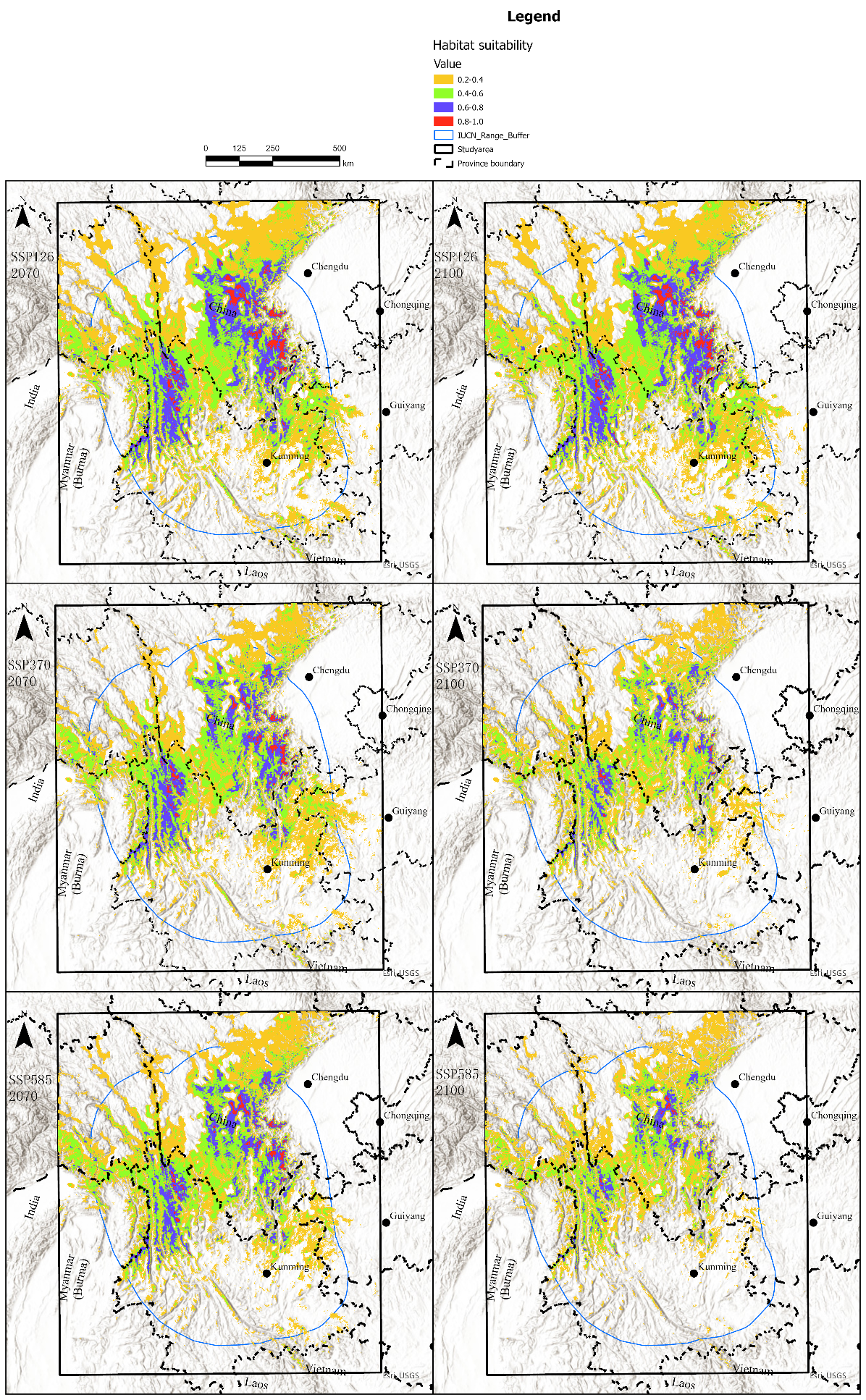


Figure S3. Future habitat suitability map of C. amherstiae under different SSP-RCP scenarios (SSP126, SSP370, and SSP585) and time periods (2041-2070, 2071-2100). Bule polygons are the 110-km buffer of IUCN range map.


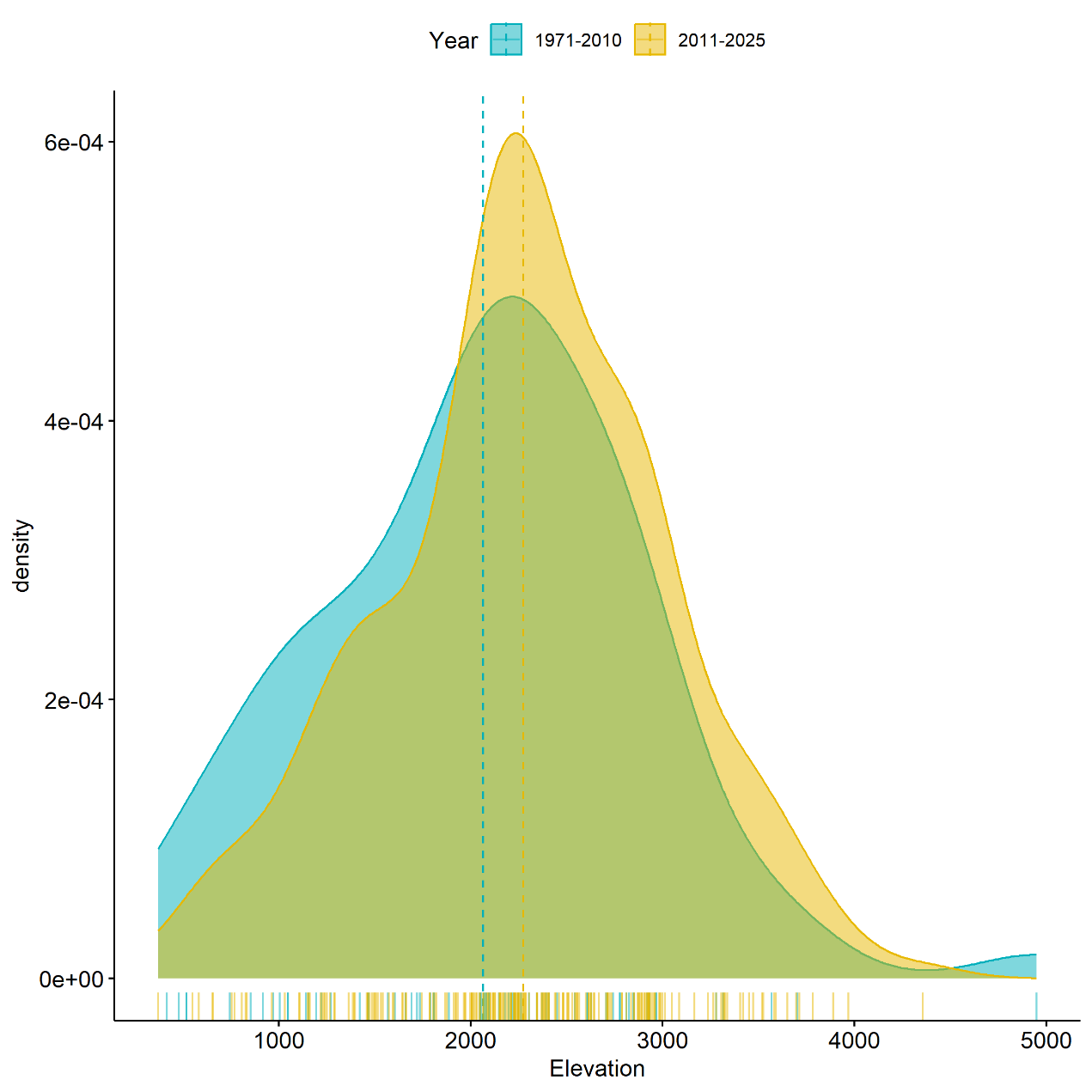


Figure S4. Density plot of elevation for two periods (1971-2010 and 2011-2025). Elevation was extracted within ArcGIS Pro based on the *C. amherstiae* occurrence points that downloaded from the GBIF.
